# Tips and tricks in tremor treatment

**DOI:** 10.1007/s00702-024-02806-x

**Published:** 2024-07-24

**Authors:** Franziska Hopfner, Carsten Buhmann, Joseph Classen, Florian Holtbernd, Stephan Klebe, Jiri Koschel, Zacharias Kohl, Sebastian Paus, David J. Pedrosa

**Affiliations:** 1 Department of Neurology, Neurologische Klinik und Poliklinik mit Friedrich Baur Institut, Ludwig-Maximilians University, Campus Großhadern, Marchioninistraße 15, 81377 Munich, Germany; 2 Department of Neurology, University Medical Center Hamburg-Eppendorf, Hamburg, Germany; 3 Department of Neurology, Leipzig University Medical Center, Liebigstraße 20, 04103 Leipzig, Germany; 4 Department of Neurology, RWTH Aachen University, Pauwelsstraße 30, Aachen, Germany; 5 JARA-BRAIN Institute Molecular Neuroscience and Neuroimaging, Juelich Research Center GmbH and RWTH Aachen University, Aachen, Germany; 6 Department of Neurology, Essen University Hospital, 45147 Essen, Germany; 7 Department of Neurology, Knappschaftskrankenhaus Recklinghausen, Recklinghausen, Germany; 8 Parkinson-Klinik Ortenau, GmbH & Co KG, Kreuzbergstraße 12-16, 77709 Wolfach, Germany; 9 Department of Neurology, University of Regensburg, Regensburg, Germany; 10 Department of Neurology, GFO Clinics Troisdorf, Troisdorf, Germany; 11 Department of Neurology, Philipps University Marburg, Marburg, Germany; 12 Centre for Mind, Brain and Behaviour, Philipps University Marburg, Marburg, Germany

**Keywords:** Tremor, Tremor treatment

## Abstract

Tremor, whether arising from neurological diseases, other conditions, or medication side effects, significantly impacts patients' lives. Treatment complexities necessitate clear algorithms and strategies. Levodopa remains pivotal for Parkinson's tremor, though response variability exists. Some dopamine agonists offer notable tremor reduction targeting D2 receptors. Propranolol effectively manages essential tremor and essential tremor plus (ET/ET +), sometimes with primidone for added benefits, albeit dose-dependent side effects. As reserve medications anticholinergics and clozapine are used for treatment of parkinsonian tremor, 1-Octanol and certain anticonvulsant drugs for tremor of other orign, especially ET. Therapies such as invasive deep brain stimulation and lesional focused ultrasound serve for resistant cases. A medication review is crucial for all forms of tremor, but it is particularly important if medication may have triggered the tremor. Sensor-based detection and non-drug interventions like wristbands and physical therapy broaden diagnostic and therapeutic horizons, promising future tremor care enhancements. Understanding treatment nuances is a key for tailored tremor management respecting patient needs and tolerability. Successful strategies integrate pharmacological, non-invasive, and technological modalities, aiming for optimal symptom control and improved quality of life.

## Introduction

Tremor, an involuntary, rhythmic muscle contraction leading to shaking movements in one or more parts of the body, is a common neurological symptom observed in the general population. Its prevalence varies depending on age, underlying health conditions, and the methodology used in different studies. Epidemiological studies suggest that tremor affects a significant portion of the general population. A review by Louis and Ferreira (2010) found that essential tremor (ET), the most common type of tremor, affects approximately 0.4% of individuals globally, with a higher prevalence in older adults (Louis and Ferreira 2010). Similarly, a study by Louis et al. (2009) highlighted that the prevalence of ET in individuals aged 65 and older is about 4.6%, indicating a marked increase with advancing age. (Louis et al. 2000)

Additionally, the incidence of Parkinson's disease, which often includes tremor as a prominent symptom, also rises with age. According to a study by de Lau and Breteler (2006), the prevalence of Parkinson's disease increases from about 0.3% in those aged 60–69 years to 2.6% in those aged 80 and older (Lau and Breteler 2006). This underscores the broader trend that neurological conditions associated with tremor are more common in the elderly.

n this review, we will discuss the treatment of the most common tremor syndromes that we encounter in everyday clinical practice. In addition to the standard treatment regimes, we will provide additional tips and tricks to help you treat your patients in a satisfactory manner.

W will explore a comprehensive array of therapeutic approaches, from medication to invasive techniques, and non-drug interventions. Pharmacological standard treatment regimens are discussed taking into account the treatment of predominantly elderly patients.. Additionally, we highlight the role of reserve medications like clozapine and certain anticonvulsants for other tremor origins.

For patients unresponsive to medication, invasive therapies like deep brain stimulation and lesional focused ultrasound are evaluated, providing a further treatment achieving excellent results in selected patients. Beyond these, we assess non-drug treatments involving peripheral non-invasive medical devices and physical therapy. There is growing evidence that sensor-based tremor detection further enhances diagnostic and treatment capabilities, promising improved patient outcomes.

Our review is based on the recent "Tremor Leitlinie" and "Parkinson Leitlinie" of the Deutsche Gesellschaft für Neurologie, which are the result of an intense and structured review of the literature (Henderson and Yiannikas 1994; Tedeschi and Sasso 1990). All evaluations were based on existing clinical studies and evidence of statistical superiority of the intervention relative to placebo or a comparator drug. For some of the tremor types discussed, specific severity levels were used in the studies. The most studied type of tremor is essential tremor, which can be measured using the established Fahn-Tolosa-Marin Tremor Rating Scale (FTMTRS) or the TETRAS scale (Ondo et al. 2018). Parkinson's tremor is usually assessed using only the tremor items of the Unified Parkinson's Disease Rating Scale (UPDRS) (Pirker and Katzenschlager 2023). Newer, more specific scales are available for Parkinson's tremor and orthostatic tremor, but have not yet been incorporated into larger clinical studies. There is no clinical calculation of a minimum clinical improvement threshold for different types of tremor. Therefore, the LL group agreed with the following statement: A 40% improvement in clinical and/or accelerometric measures is considered a clinically relevant improvement in tremor. If quality of life measures were available, these would be included in the assessment. However, specific thresholds for these measures could not be established. Additionally, the authors have conducted further literature searches to incorporate their individual recommendations, providing valuable insights into the daily clinical routines of movement disorder experts.

This review, while aligning with international recommendations for tremor treatment, is strongly guided by the German guidelines (Ferreira et al. 2019; Bhatia et al. 2018; Olanow et al. 2018), providing a detailed guide for tailored tremor management, integrating pharmacological, non-invasive, and technological modalities to optimize symptom control and improve patients' quality of life.

### Levodopa for tremor treatment

Levodopa is the most efficacious treatment for rigidity, bradykinesia and tremor in Parkinson's disease (PD) (Fahn et al. 2004). Of the three cardinal symptoms, tremor responds less consistently to this drug than rigidity and bradykinesia (Luo et al. 2019). Several trials have shown the positive effect of levodopa on rest tremor (Koller 1986). One study demonstrated an effect of Levodopa on postural PD tremor (Henderson et al. 1994). Koller et al. found a 55% reduction of the tremor amplitude in PD with levodopa (Koller 1986). PD tremor was significantly reduced compared to placebo after one-time treatment with 250 mg levodopa (with carbidopa) in three clinical trials (Henderson et al. 1994; Tedeschi et al. 1990; Hughes et al. 1990). The primary approach to reduce PD tremor should follow the same principles as the treatment of the other levodopa-responsive motor features. Thus, for each patient the adequate levodopa-dose has to be determined individually, considering all symptoms. In some PD patients tremor does not respond to levodopa at all (Pirker et al. 2023). The less consistent effect of levodopa on tremor likely reflects different pathophysiological mechanisms from akinesia and rigidity. (Pirker et al. 2023)

To our knowledge, no studies have been conducted to determine whether an increase of the dose of levodopa may be beneficial in patients experiencing poor tremor control with standard dosages sufficient to control bradykinesia. This may be due to the wide variety of motor response to levodopa and the lack of tolerability of higher doses of dopaminergic drugs in PD patients. If patients treated with standard doses of levodopa report a disturbing tremor, levodopa should be increased while monitoring side effects. As standard dose for newly diagnosed PD we would recommend 3 times 100 mg levodopa (with decarboxylase inhibitor), if symptoms are not sufficient controlled with 3 × 50 mg or 75 mg levodopa per day. If PD tremor does not improve at 3 times of 150 mg levodopa, other therapeutic options should be taken into account. In young patients who are naive to levodopa, treatment with dopamine agonists and MAO-B inhibitors can be considered first and foremost due to the risk of motor complications caused by levodopa. As the disease progresses, PD-symptoms are often hampered by the occurrence of motor fluctuations associated with levodopa treatment (Cilia et al. 2014). All variations of PD tremor can show improvement in On-state and deterioration in Off-state as shown in a study by Gupta et al. (Gupta et al. 2020). Therefore, the therapeutic aim of levodopa responsive tremor should be optimizing the current dopaminergic stimulation by increasing the strength of each dose or give levodopa more frequently, without causing side effects and adding MOA-B inhibitors and/or COMT inhibitors. The next step to improve tremor symptoms and motor fluctuations in PD would be a continuous intestinal infusion of levodopa/carbidopa gel (Olanow et al. 2014) or subcutaneous infusion of foslevodopa/foscarbidopa (Aldred et al. 2023), if the patients are not eligible for deep brain stimulation (DBS). It has to be mentioned that PD tremor, as all tremors, can be amplified by cognitive stress (Zach et al. 2017). If tremor is responsive to levodopa, patients could try soluble levodopa or inhaled levodopa on demand if they expect or suffer stressful situations with intensified tremor. In advanced PD, the response to regular doses of levodopa sometimes is unreliable, potentially leading to partial-ON, delayed-ON, or no-ON responses (Olanow et al. 2021). In these situations, levodopa on demand may also be helpful to ease PD tremor. Careful monitoring of on demand levodopa therapy is recommended to avoid the emergence of a dopamine dysregulation syndrome (inadequate and compulsive intake of levodopa to avoid the unfavorable effects of the withdrawal of dopamine replacement therapy) (O'Sullivan et al. 2009). Increasing the overall levodopa daily dosage may be beneficial if on demand therapy is required regularly (Sasikumar et al. 2021). Overall, keeping a diary documenting the timing of restrictive tremor symptoms over several weeks can be helpful in guiding treatment.

### Dopamine-agonists for tremor treatment

Dopamine receptors play a crucial role in various physiological and pathological processes, including motor control. The diversity in binding affinities of dopamine agonists to different dopamine receptors leads to varied functional expressions in experimental models and patients, with both beneficial and detrimental effects (Millan 2010; Millan et al. 2022). Dopamine agonists interact with a range of receptors beyond dopamine receptors, including serotonergic and adrenergic receptors (Millan 2010; Millan et al. 2022; Newman-Tancredi et al. 2002). Dopamine receptors influence intracellular signaling pathways through their seven antiparallel transmembrane domains. Historically, the discovery of dopamine subreceptors, particularly D1 and D2 receptors, shaped the understanding of motor complications associated with levodopa therapy in PD. Co-stimulation of both receptors was considered necessary for symptom management. (Gershanik et al. 1983; Beaulieu 1987) Subsequent cloning efforts identified five dopamine subreceptors (D1-D5) by the late 1980s. (Bunzow et al. 1988; Seeman 2015) Recent structural studies have elucidated the binding behavior of dopamine agonists to specific receptors like D2, D3, and D4 receptors, contributing to drug development and understanding binding modes (Chien et al. 2010; Wang et al. 2001, 2017, 2018) Differences in genetic origin divide dopamine receptors into D1-like (D1, D5) and D2-like (D2, D3, D4) receptors, each with distinct biological effects mediated through different intracellular signaling pathways (Alexander et al. 2021; Hilger et al. 2018). The role of dopamine receptors in PD treatment is multifaceted. Genetic polymorphisms in D2 receptors influence therapy response, suggesting their involvement in PD pathogenesis (Masellis et al. 2016; Tinsley et al. 2009). Decreasing D2 receptor density with age correlates with cognitive deficits. (Holstein et al. 2011)

The choice of dopamine agonists for PD treatment may be guided by their receptor affinities, which determine their clinical effects. Drugs like pramipexole and ropinirole, which act primarily on D2 receptors, have been developed based on this understanding (Wilson et al. 2020). Dopamine agonists with high D2/D3 receptor affinity show a strong quantitative effect on tremor, in individual cases even higher than the highest doses of levodopa. (Bartl et al. 2022; Luo et al. 2022; Sheng et al. 2021; Zou et al. 2022)

### Propranolol for tremor treatment

Propranolol as non-selective beta-blocker is superior to other beta-blockers in the treatment of tremor and is applied broadly across different forms of tremor (Tables 1, 2) (Hopfner et al. 2020). The proposed mechanism of action is by blocking peripheral non-cardiac beta-2 receptors located in muscle spindles. (Hedera 2017; Hopfner and Deuschl 2020)

**Table 1:** Overview of the tremor syndromes their tremor characteristics and Medication for treatments

Tremor syndrome	Tremor frequency (Hz)	Pathognomonic characteristics	Medication for treatment
Essential tremor	4–12 Hz	Bilateral, symmetric tremor primarily affecting the hands, may also involve the head, voice, and legs; improves with alcohol. ET Plus:	Propranolol, Primidone, Topiramate, Gabapentin, Botulinum Toxin
ET Plus (ET +)	4–12 Hz	Additional Neurological Signs: ET + is characterized by the presence of additional neurological signs that are not seen in pure essential tremor. These can include Mild gait disturbances, Subtle cognitive changes, Mild dystonia, Mild ataxia	Propranolol, Primidone, Topiramate, Gabapentin, Botulinum Toxin
Parkinsonian tremor	4–6 Hz	Mainly Resting tremor, "pill-rolling" character, decreases with voluntary movement, often asymmetrical, associated with bradykinesia and rigidity. Postural tremor, e-emergent tremor, which occurs in approximately two-thirds of patients	Levodopa, Carbidopa, Dopamine agonists, Anticholinergics Amantadine, Propranolol
Cerebellar tremor	< 5 Hz	Intention tremor, worsens with purposeful movement, often accompanied by dysmetria, dysarthria, and gait ataxia	Propranolol, Primidone, Clonazepam, Topiramate, Gabapentin, Botulinum Toxin
Physiological tremor	8–12 Hz	Fine tremor, usually not visible, enhanced by anxiety, fatigue, caffeine, and certain medications	Addressing underlying cause (e.g., thyroid disorders, medication adjustments), Beta-blockers (Propranolol)
Dystonic tremor	Variable	Occurs in body parts affected by dystonia, irregular and jerky, often positional and can be relieved by specific sensory tricks	Botulinum Toxin, Anticholinergics (Trihexyphenidyl), Baclofen, Benzodiazepines in individual patients
Orthostatic tremor	13–18 Hz	Rapid, rhythmic muscle contractions, primarily in the legs when standing, improves when sitting or walking	Clonazepam, Gabapentin, Valproic Acid, Levodopa, Phenobarbital
Drug-induced tremor	Variable	Can be similar to physiological or parkinsonian tremor, depends on the causative drug (e.g., beta-agonists, antipsychotics, lithium)	Reducing or discontinuing causative drug, Beta-blockers (Propranolol)
Rubral tremor	2–4 Hz	Combination of rest, postural, and intention tremor, typically caused by lesions in the brainstem or cerebellum	Levodopa, Clonazepam, Propranolol, Topiramate
Psychogenic tremor	Variable	Variable frequency and amplitude, abrupt onset and remission, distractibility, often associated with psychiatric conditions	Addressing underlying psychological issues, Cognitive-behavioral therapy, Antidepressants (SSRIs) in individual cases
Holmes tremor\rubral tremor	2–4 Hz	Irregular, low-frequency tremor occurring at rest, posture, and action; often associated with brainstem or thalamic lesions	Levodopa, Clonazepam, Propranolol, Trihexyphenidyl, Gabapentin

**Table 2:** Use of propranolol for different tremor subtypes according to the Guidelines of the German Neurological Society (Deuschl 2022)

Tremor	Specific recommendation regarding propranolol
Essential tremor and Essential tremor plus (Hopfner and Deuschl 2018; Chen et al. 2017)	Among the drugs of first choice for ET is propranolol. (Hopfner and Deuschl 2020) Daily doses of 30–320 mg (mostly 60–240 mg) are recommended. (Hopfner and Deuschl 2020) Low daily dosages (30–60 mg) can be tried for small amplitude tremors
Increased physiological tremor	Propranolol cannot be generally recommended for the treatment of increased physiological tremor, but can be considered in individual cases, taking into account the side effect profile
Focal tremor	The standard drugs propranolol, primidone and topiramate are less effective for essential head tremor than for hand tremor—albeit with insufficient studies. In dystonic head tremor, treatment with propranolol can be dispensed with. The efficacy of propranolol and primidone has not been sufficiently proven in voice tremor due to insufficient studies. However, as the alternative interventions are all invasive, these drugs can be tried in individual cases
Dystonic tremor	In dystonic hand tremor, treatment with propranolol is not considered helpful. Propranolol or primidone cannot be generally recommended for the treatment of task-specific hand tremor, but can be considered in individual cases
Parkinson's tremor	Beta-blockers can be considered for the treatment of postural Parkinson's tremor (Off-label)
Cerebellar tremor	Treatment with propranolol can be considered in individual cases despite a lack of evidence
Orthostatic tremor	In patients with orthostatic tremor, therapy with pramipexole, primidone, propranolol or valproate can be dispensed with

In general, propranolol is well tolerated. If side effects occur, the dose should not be increased further. Propranolol should not be used if severe bradycardia or second or third degree atrioventricular block are present. Side effects include hypotension, fatigue, depression, and erectile dysfunction. Propranolol is contraindicated in patients with asthma and should be used with caution in patients with diabetes because of its masking effect on hypoglycemia (Sharma and Pandey 2019). In general, patients are advised to increase their medication slowly by increasing the dose by 20 mg propranolol per week and taking propranolol three times a day. A dosage increase to 320 mg takes 4.5 months with a weekly increase of 20 mg.

Some patients prefer to not take propranolol continuously but rather as needed, as temporary relief from tremor is sufficient for them (for example when giving a public speech). Although this works well for some patients, this approach to medication should be carefully considered. Short-term improvements may vary between 30 and 75% (average 44%) as assessed by accelerometric measurements. Long-term experience is limited and based on uncontrolled case series. (Deuschl et al. 2011)

### Primidone for tremor treatment

Primidone has initially been introduced as an anti-seizure medication. Primidone is rapidly metabolized to phenobarbital and phenylethylmalonamide (Lenkapothula and Primidone 2024). Primidone and its metabolite phenobarbital act by increasing GABAergic inhibition at the barbiturate binding site of the GABAA receptor. (Lenkapothula and Primidone 2024)

### Primidone for essential tremor

Primidone is effective in the treatment of ET and ET + , but only about 50% of patients are responsive to therapy (Deuschl et al. 2011). Primidone has no effect on tremor frequency, but reductions of tremor amplitude by an average of 50–60% have been reported in most studies. (Deuschl et al. 2011) Several reports have confirmed long term efficacy of primidone, and low dose regimens (250 mg per day) seem to be equally effective compared with higher doses (Serrano-Duenas 2023; Koller and Vetere-Overfield 1989; Koller and Royse 1986) However, there also is evidence for a loss of treatment efficacy and habituation over time in some individuals. (Sasso et al. 1990; Crystal 1986)

Side effects often limit the usage of primidone. There is a clear dose dependency, and adverse effects are common (Serrano-Duenas 2023; Koller and Royse 1986; Findley et al. 1985). Of note, most patients discontinue treatment within the first 3 months, whereas long-term tolerance on stable doses usually is good (Serrano-Duenas 2023; Koller and Vetere-Overfield 1989). Many patients complain about an initial toxicity of primidone characterized by nausea, dizziness, ataxia, unsteady gait, and sedation within the first days after treatment (Koller and Vetere-Overfield 1989). In contrast to long-term treatment, a previous study did not find an effect of dose on the occurrence or severity of initial toxicity (O'Suilleabhain and Dewey 2002). That said, in our experience, it is wise to start primidone at very low doses to improve adherence to therapy. In Germany, only 250 mg tablets are available, resulting in a minimal starting dose of 62.5 mg (1/4 tablet). If no tablet formulation with a lower dose is available, we recommend using primidone suspension. We typically initiate therapy at 25 mg suspension at bedtime with increments of 25 mg every 72 h until a sufficient symptom control or a dose of 250 mg is reached. Then, switching to the tablet formulation is recommended, and further dose increments should be divided in 2–3 single doses per day. In our experience, it is not meaningful to increase the daily dose above 500 mg if there is no treatment response. Interestingly, primidone appears to be tolerated better by patients treated for epilepsy that often receive much higher doses (Lenka and Louis 2021). The reasons for this discrepancy are unknown. Epilepsy patients on average are younger than ET patients, which imply an impact of age on tolerability.

Along these lines, most experts prefer propranolol to primidone in younger ET individuals. However, there is insufficient data to confirm this hypothesis, and available studies do not suggest that age has a relevant impact on primidone tolerability in ET (Findley et al. 1985). It is not uncommon for ET patients to develop additional motor and non-motor symptoms during the course of the disease, including ataxia and cognitive decline (Louis 2023). These symptoms have similarity with primidone toxicity and may decrease patient’s tolerability of primidone. Lastly, anticonvulsive co-medication may improve primidone tolerance in epilepsy patients (“cross-tolerance”) (Kanner et al. 2000). ET is a heterogeneous disease, as is its response to therapy with primidone. Whereas some patients show an excellent treatment effect, others do not, and tolerability and long-term efficacy differ substantially. Despite being recommended as standard therapy prescription of primidone still is “Off-label” for the treatment of ET in Germany (Deuschl et al. 2011). There are no large blinded studies on the use of primidone in other tremors. Primidone plays a subordinate role in the treatment of other tremors.

#### Primidone for dystonic tremor

Several studies have investigated the efficacy of primidone in treating dystonic tremor. A study by Jankovic and Fahn (1986) included patients with various forms of tremor, including dystonic tremor, and found that primidone significantly reduced tremor severity in a majority of cases (Jankovic and Fahn 1986). Patients with dystonic tremor reported improvements in tremor amplitude and functional abilities, suggesting that primidone can be an effective treatment option. Additionally, a retrospective study by examined the long-term outcomes of patients with dystonic tremor treated with primidone (Fasano et al. 2014). The study reported that a substantial proportion of patients experienced sustained tremor relief over several years, with tolerable side effects. This finding supports the potential long-term benefits of primidone for managing dystonic tremor. Primidone has been compared with other medications, such as propranolol and botulinum toxin, in the treatment of dystonic tremor. In a study by Espay et al. (2018), primidone was found to be effective in reducing tremor severity, although botulinum toxin was often preferred due to its targeted action and fewer systemic side effects (Espay et al. 2017). However, primidone remains a valuable option, especially for patients who do not respond to or cannot tolerate other treatments. As with treatment of ET, side effects are common, including sedation, dizziness, and ataxia. These side effects can often be minimized by starting at a low dosage and gradually titrating the dose (see section on primidone in the treatment of ET above). Long-term use of primidone has been associated with the development of tolerance, necessitating periodic reassessment of its efficacy and dosage adjustments. Primidone is a viable treatment option for dystonic tremor, offering significant relief for many patients. Its effectiveness in reducing tremor severity and improving functional outcomes makes it a valuable tool in the management of this challenging condition. However, as with any medication, its use should be tailored to the individual patient, considering both the benefits and potential side effects.

### Propranolol + Primidone: additive effect for tremor treatment

An additive effect has been shown for propranolol and primidone in ET and ET + . The selection of the individually suitable intervention is based on contraindications and individual tolerability. The combination of primidone (250 mg) and propranolol (80 mg) is more effective than each treatment on its own. (Deuschl et al. 2011)

### Clozapine for tremor treatment

Clozapine, an atypical antipsychotic drug, is not typically used as a first-line treatment for PD tremor. However, it has been studied for its potential benefits in managing tremors associated with PD, particularly when other medications have not been effective, have caused intolerable side effects, or the PD patient is not eligible for DBS. In PD clozapine serves as first line therapy in severe PD associated psychosis (Seppi et al. 2019). A positive influence of clozapine on tremor in PD patients was already described at the end of the last century (Pfeiffer and Wagner 1994). Regarding the unique pharmacological profile of clozapine, it may exert beneficial effects on tremor in PD through its antagonistic action at some dopamine receptor subtypes. However, unlike typical antipsychotics, clozapine has a lower affinity for D2 receptors, which are involved in the regulation of motor function. Moreover, clozapine's affinity for other neurotransmitter receptors, such as serotonergic, cholinergic, adrenergic, and histaminergic receptors may contribute to its anti-tremor effects (Yaw et al. 2016). Clinical studies investigating the efficacy of clozapine in PD-related tremor has shown promising results. Several open label studies and, to our knowledge, two randomized controlled trials have investigated the effect of clozapine on tremor, particularly in patients who were refractory to other pharmacological interventions (Yaw et al. 2016; Bonuccelli et al.^1997^; Friedman et al. 1997). Yet, the treatment of PD tremor with clozapine is still “Off-label”. It is essential to consider the potential risks associated with clozapine therapy, including hematological abnormalities and agranulocytosis (Yaw et al. 2016), which necessitate regular monitoring. Furthermore, individual patient characteristics, such as disease stage, medication history, and comorbidities, should be carefully evaluated when considering clozapine as a treatment for PD-related tremor. The dosage of clozapine for PD tremor can vary depending on individual factors such as the severity of symptoms, tolerance to the medication, and any concurrent medical conditions. Generally, when used for PD tremor, the dosage of clozapine is lower compared to its dosage for treating schizophrenia. Gradual adjustment of dosage (commencing at either 6.25 or 12.5 mg), administering a single dose at bedtime, or considering a higher clozapine dose at night and a minimal morning dose could enhance patient tolerance in cases where sedation or orthostatic hypotension are observed in response to treatment. In the two randomized controlled trials (Bonuccelli et al. 1997; Friedman et al. 1997) the mean clozapine dosage to treat PD tremor was 45 ± 9.6 mg per day for a period of 15.5 ± 8.3 months (Bonuccelli et al. 1997) and 39 mg/day over six weeks (Friedman et al. 1997) respectively. Since both randomized studies are > 20 years old further research and clinical trials are needed to elucidate the long-term efficacy and safety of clozapine in this context and to establish clear guidelines for its use in PD management.

### 1-octanol for tremor treatment

Ethanol has been reported to improve tremor severity in approximately two thirds of patients with ET (McGurrin et al. 2024; Knudsen et al. 2011; Hopfner et al. 2015). However, the response of a tremor to alcohol is not specific to one form of tremor. In addition to patients with ET, a non-significant proportion of patients affected by Parkinson's tremor and dystonic tremor report an improvement in tremor amplitude as a result of alcohol intake (Mostile and Jankovic 2010; Junker et al. 2018). Octanol is a fatty alcohol, has garnered interest due to its ability to modulate neurotransmitter activity in the brain, leading to tremor reduction. 1-octanol has been shown to act as a GABAergic agonist, influencing the inhibitory neurotransmitter gamma-aminobutyric acid (GABA) in the brain (Kurata et al. 1999). GABAergic mechanisms play a crucial role in regulating neuronal excitability, and disturbances in GABAergic signaling are implicated in various movement disorders, including tremor (Haubenberger et al. 2014; Ondo 2022). By enhancing GABAergic transmission, 1-octanol may help restore the balance between excitatory and inhibitory signals in the brain, thereby reducing tremor severity. (Ondo 2022)

Several clinical studies have explored the efficacy of 1-octanol in tremor management, particularly in ET patients where traditional therapies like beta-blockers or anticonvulsants such as pregabaline, gabapentine may not provide adequate relief.

Several studies have shown significant reduction in tremor amplitude, with minimal adverse effects reported. This initial study laid the foundation for further investigation into octanol's therapeutic benefits. (Haubenberger et al. 2014; Bushara et al. 2004; Nahab et al. 2011; Voller et al. 2016)

### Topiramate for tremor treatment

Topiramate, an anticonvulsant medication commonly used for epilepsy and migraine prevention, has shown promise in the treatment of tremor, particularly ET. Its mechanism of action involves modulating voltage-gated sodium channels, enhancing GABAergic inhibition, and antagonizing AMPA/kainate glutamate receptors. This multifaceted pharmacological profile makes it a candidate for managing tremor symptoms. Several studies have explored the efficacy of topiramate in tremor treatment. A randomized, double-blind, placebo-controlled trial demonstrated the effectiveness of topiramate in reducing tremor severity in patients with essential tremor. Patients receiving topiramate showed significant improvement in tremor ratings compared to those on placebo (Connor et al. 2008). Another study evaluated the long-term use of topiramate in ET patients. Results indicated that topiramate maintained its efficacy over an extended period, with a good safety and tolerability profile (Chang et al. 2015). In a study focusing on the comparative effects of different anticonvulsants on ER, topiramate was found to be as effective as other treatments such as gabapentin, with a favorable side effect profile (Galvez-Jimenez and Hargreave 2000). An open-label trial assessed topiramate in patients with ET and Parkinsonian tremor (Connor et al. 2008; Shah et al. 2022). The study concluded that topiramate could be a viable option for patients who do not respond to propranolol and primidone (Zesiewicz et al. 2011). Overall, topiramate appears to be a valuable addition to the pharmacological arsenal against tremor. It offers an alternative for patients who may not respond adequately to traditional medications or who experience intolerable side effects. The studies mentioned provide a solid foundation for considering topiramate as a treatment option, demonstrating its efficacy and tolerability in clinical practice. However, doses of > 200 mg are often required for a satisfactory therapeutic effect on tremor amplitude.

### Other antiepileptic drugs for tremor treatment

No significant benefit has been demonstrated for levetiracetam, gabapentine and pregabalin. (Ferreira et al. 2019)

### Benzodiazepines for tremor treatment

Benzodiazepines, are generally not recommended for the treatment of tremor irrespective of the underlying cause: the use is recommended in individual cases only. Therapeutic effects have been reported for orthostatic tremor, dystonic tremor, and tremors associated with anxiety reducing tremor amplitude and improving motor function (Davis et al. 1995; Rajput and Rajput 2014; Biary and Koller 1987). The most significant concerns include the development of tolerance, dependence, and other adverse effects. Common side effects of benzodiazepines include drowsiness, dizziness, and impaired coordination, which can impact daily functioning and increase the risk of falls, particularly in the elderly. Cognitive impairment and memory issues are also concerns with long-term use. Benzodiazepies can interact with other medications, potentially leading to enhanced side effects or reduced efficacy of other treatments. Patients taking other CNS depressants or medications metabolized by the same liver enzymes need careful management to avoid adverse interactions.

#### *Cannabis*-based medication for tremor treatment

Cannabinoids, specifically medical cannabis derived from the Cannabis plant, have gained attention in recent years for its potential therapeutic benefits in managing various medical conditions, including tremor. Cannabinoids interact with the endocannabinoid system in the body, which plays a crucial role in regulating various physiological processes, including motor control and neurotransmitter release. Delta-9-tetrahydrocannabinol (THC) and cannabidiol (CBD) are two well-known cannabinoids with distinct mechanisms of action (Chayasirisobhon 2020). THC primarily binds to cannabinoid receptors in the brain, particularly CB1 receptors, leading to psychoactive effects but also modulating neurotransmitter release and neuronal excitability (Chayasirisobhon 2020). CBD, on the other hand, interacts with multiple receptors and neurotransmitter systems, exerting anti-inflammatory and anxiolytic effects without the psychoactive properties of THC. (Chayasirisobhon 2020)

The results on the effect of cannabis-based medications are not consistent with regard to treatment effect on tremor (Smith and Wagner 2014; Shahsavar et al. 2018; Santos de Alencar et al. 2021). There is a lack of standardized clinical trials to validate the efficacy and safety of cannabis-based medications on tremor severity and patients' well-being. However, it can be concluded that an anxiolytic effect can reduce tremor, especially tremor in the context of PD. (Faria et al. 2020; Chagas et al. 2014)

### Botulinum toxin (BoNT) therapy for tremor treatment

Botulinum toxin (BoNT) therapy has emerged as a valuable treatment option for various types of tremor, particularly for dystonic tremor. BoNT injections have demonstrated efficacy in reducing the severity of dystonic tremor and improving patients' quality of life. Several studies have investigated the effectiveness of BoNT therapy for dystonic tremor, especially in the upper limbs (Anandan and Jankovic 2021). For instance, a meta-analysis reported a significant reduction in tremor severity following BoNT injections in patients with upper limb tremor associated with dystonia (Mittal et al. 2019; Simpson et al. 2008). The effect size of BoNT treatment on tremor reduction was found to be clinically meaningful, with improvements sustained over several weeks to months.

Moreover, BoNT therapy has been shown to provide long-lasting benefits for tremor management. While the duration of treatment efficacy may vary depending on individual patient factors and the specific type of tremor, studies have consistently reported sustained tremor reduction for several months following BoNT injections (Pappert and Germanson 2008). Emerging evidence suggests its potential utility in other tremor disorders, such as ET and Parkinsonian tremor. Carefully conducted studies for these indications are, however, lacking. (Anandan and Jankovic 2024; Jankovic and Schwartz 1991)

### Calcium channel blockers for tremor treatment

Calcium channel blockers (CCBs) have been investigated in the treatment of tremor.The mechanism of action underlying the tremor-suppressing effects of CCBs is thought to involve their ability to inhibit calcium influx into neurons, thereby reducing neuronal excitability and neurotransmitter release. By modulating calcium channels, CCBs may help regulate the abnormal oscillatory activity observed in tremor circuits, leading to tremor suppression. Several studies have investigated the efficacy of CCBs, particularly nimodipine and isradipine, in the management of tremor (Biary et al. 1987; Zesiewicz et al. 2013). Convincing evidence of tremor reduction could not be provided for CCBs. Further research is needed to elucidate their optimal dosing regimens, long-term efficacy, and safety profiles. Additionally, large-scale RCTs comparing CCBs to standard ET treatments are warranted to establish their role in clinical practice.

### Invasive and lesional thesecrapies for tremor treatment: deep brain stimulation

The treatment of tremor with invasive methods originated in the 1950s when lesions in the thalamus were developed using radiofrequency heating (Gardner 2013). The principle of targeted destruction or electrical modulation of parts of the tremor loop in the central nervous system has been further developed and applied in various core areas, including those relevant to tremor treatment.

Deep brain stimulation (DBS) is an established procedure for the treatment of patients with PD who have motor fluctuations and dyskinesia that cannot be treated with medication or a tremor that cannot be controlled with medication, with the majority of DBS performed in the subthalamic nucleus (STN) (Okun 2012). However, alternative anatomical target points such as thalamic nuclei as ventral-intermediate-nucleus; (VIM), globus pallidus internus (GPI), N. ventralis oralis anterior (VOA) can also be considered in tremor therapy in PD. Bilateral electrical stimulation of the STN should be considered in PD patients with insufficient medical treatment because of its efficacy on all cardinal motor symptoms including tremor, motor fluctuations and dyskinesia Bilateral GPI should be considered as alternative target in PD patients with borderline cognition or age, especially when there is no strict need for reduction of medication dosage postsurgically. It is crucial to weigh the special risks associated with the surgical procedure against the therapy's benefits. (Deuschl and Krack 1996)

For patients with PD and contraindications to STN stimulationbesides GPI, unilateral or bilateral thalamic stimulation can be considered as a treatment option (Okun 2012). Randomized, controlled studies comparing DBS with best medical treatment (BMT) are available only for STN and GPI DBS (Weaver et al. 2009). A review of all randomized trials comparing STN vs GPI DBS in PD showed a medium effect size (0.36) in reducing tremor without significant difference between both targets. Tremor suppression in STN-DBS shows more fluctuations over time compared to GPI-DBS (Wong et al. 2019). Additionally, non-randomized studies have shown substantial improvement in resting and action tremor with STN DBS, with improvements ranging from 78 to 82% in different tremor types after one to twelve months (Williams et al. 2010; Krack et al. 1998). Long-term studies over 5 to 10 years also indicate a mean improvement in resting tremor post-STN DBS. (Benabid et al. 1991)

Notably, there is a lack of randomized, controlled studies directly comparing the efficacy of STN DBS versus thalamic DBS in PD tremor, highlighting a need for further research in this area to guide treatment decisions effectively. (Hubble et al. 1997)

In tremor syndromes other than PD, DBS of the posterior-subthalamic-area (PSA), caudal zona incerta (cZi) or VIM are used to treat medication-resistent tremor,

Unilateral thalamic DBS should be offered to severely affected patients with medication-resistant essential tremor ET. Bilateral thalamic stimulation shows better efficacy than unilateral stimulation in retrospective studies, especially for head and voice tremor (Deuschl and Krack 1996). Therefore, bilateral DBS should be considered for patients with severe axial tremor (additional or pure severe voice and/or head tremor) if medication therapies are inadequate (Deuschl and Krack 1996). However, randomized comparative studies between bilateral and unilateral stimulation, as well as between DBS outside the STN or GPI and medication treatment, are lacking (Deuschl and Krack 1996).

Data from various studies show a mean improvement in tremor through unilateral DBS in the short term of approximately 82% for contralateral tremor and 57% for the overall tremor score (Wharen et al. 2017; Blomstedt et al. 2007, 2010; Cury et al. 2017; Fytagoridis et al. 2016; Hariz et al. 2002). However, there is a long-term reduction in effectiveness over time, possibly due to disease progression. (Tsuboi et al. 2020; Paschen et al. 2019)

DBS carries risks, including intracranial complications, and infections, sometimes even necessitating hardware removal (Engel et al. 2018). The rate of these events varies depending on the study and the chosen brain target, but overall, DBS is considered a safe treatment option (Engel et al. 2018). Stimulation-related side effects such as muscle contractions, paresthesias, and speech disturbances may occur but are usually reversible or can be minimized by adjusting stimulation parameters (Paschen et al. 2019; Engel et al. 2018)

Bilateral thalamic DBS should be offered for pronounced drug-resistant essential head tremor. Bilateral DBS of the GPI should be offered for pronounced drug-resistant dystonic head tremor (Deuschl and Krack 1996). Alternatively, VIM can be considered as a target if the tremor is more pronounced than the dystonia in dystonic tremor. (Deuschl and Krack 1996).

Bilateral DBS of the VIM should be offered for pronounced drug-resistant essential vocal tremor (Deuschl and Krack 1996).

Bilateral DBS of the GPI should be offered for pronounced drug-resistant dystonic voice tremor (Deuschl and Krack 1996). Alternatively, the VIM can be considered as a target point if the tremor is more pronounced than the dystonia. (D; Deuschl and Krack 1996).

### Invasive and lesional therapies for tremor treatment: MRI-guided focused ultrasound

MRI-guided focused ultrasound (MRgFUS) is a non-invasive lesional therapy for the unilateral treatment of drug-resistant ET and, with insufficient evidence to date, PD tremor (Zaaroor et al. 2018). Unilateral MRgFUS treatment should be offered to patients with drug-resistant ET if unilateral treatment is promising and / or an improvement in the patient's quality of life can be assumed despite only unilateral tremor reduction (Elias et al. 2013). The determination of the individual indication is reserved for the specialist centers. For the time being, bilateral focused MRgFUS should only be considered in the context of prospective studies due to the lack of studies and previous experience of severe dysarthria induced by bilateral radiofrequency thalamotomies. Although focused ultrasound was introduced only a few years ago, the quality of evidence for its treatment is the best because there is a randomized study comparing it to sham treatment (Elias et al. 2013). Reliable data currently exist only for unilateral stimulation, which showed a 70% improvement in lateralized scores compared to 3% in the sham group and a 41% improvement in total tremor scores compared to 2% in the sham group. Improvements in the four open-label studies (Zaaroor et al. 2018; Elias et al. 2013; Huss et al. 2015; Harary et al. 2019) were similar and even better than expected. Most studies target the VIM, but like with DBS or radiofrequency lesioning, some groups target the input region of the cerebellothalamic tract first described by Velasco (Gallay et al. 2016; Schreglmann et al. 2017; Velasco et al. 1972). Results are very similar for both targets.

Effects have been reported up to 2 years and even up to 4 years. (Chang et al. 2018; Meng et al. 2018; Park et al. 2019) Overall, results have remained stable for this period with a possible slight decline, and there were no additional adverse events reported.

Scores for improvements in head and voice tremor are not specifically shown, but some studies mention no improvement or only slight improvement in these midline tremors. (Zaaroor et al. 2018; Elias et al. 2013; Mohammed et al. 2018)

In a pooled analysis of therapy complications in 170 patients, severe side effects occurred in 1.7% (Deuschl and Krack 1996). Persistent paresthesias, numbness, ataxia, and balance issues were seen in 18% of patients over 12 months, mostly of mild severity (Deuschl and Krack 1996). Bleeding and infections did not occur.

With the currently available technology, only patients with a skull density ratio, a measure of skull permeability to ultrasound, above 0.35–0.4 are eligible for treatment (D'Souza et al. 2019). This measure can be calculated from routine head CT scans. Compared to DBS, focused ultrasound is likely more cost-effective. (Ravikumar et al. 2017)

### Treatment of drug-induced tremor

Diagnosing drug-induced tremor can be challenging due to factors such as delayed symptoms and its association with other types of tremor (Morgan and Sethi 2005). It is important to review the patient's medication history and assess additional clinical neurological signs, such as tremor entrainment or drug-induced parkinsonism (Morgan and Sethi 2005). Careful clinical examinations and comprehensive medical histories are especially important when multiple medications are being taken, as certain combinations may intensify tremor. Neurophysiology can be a valuable tool in diagnosing tremor associated with medication intake when the cause is uncertain (Wardt et al. 2020). There are reasonable criteria to differentiate between centrally and peripherally mediated tremor (Deuschl et al. 1996). However, there is no universally accepted way to differentiate between different forms of central tremor. Frequency is a useful classifier for cerebellar tremor, rubral tremor, and orthostatic tremor (Deuschl et al. 1996). Although maximum amplitude is found in Parkinson's disease, this parameter does not differentiate well between different tremors (Deuschl et al. 1996). Tremor waveform analysis is a promising tool for distinguishing different types of tremor. Electromyography can diagnose some rare forms of tremor (Deuschl et al. 1996). Methods of differentiating myoclonus from tremor include EEG/EMG related techniques, long-term reflexes, and polymyography.

Risk factors for drug-induced tremor include being older, male, taking multiple medications, high medication doses (including toxic levels), and using immediate-release formulations. (Morgan and Sethi 2005) When deciding how to treat drug-induced tremor in a patient, doctors should consider how it affects the patient's daily life, potential side effects (like amiodarone-induced hyperthyroidism), whether the patient is willing to take more medication or stop the medication causing the tremor, and potential alternatives. Certain medications, like β-blockers or primidone, may help relieve drug-induced tremors and should be considered, especially when there's a good reason to use the medication causing the tremor to treat the underlying condition. In the subsequent section, we will elaborate on medications causing tremor, along with their significant clinical implications.

Amiodarone, an antiarrhythmic medication, may induce oscillatory and involuntary movements, predominantly in forms of postural and intentional tremors at 6 to 10 Hz frequency (Table 3) (Charness et al. 1984). Metaanalyses estimate its prevalence at 5% of patients on amiodarone at 200 mg with higher odds with higher treatment dosage and longer duration (Orr and Ahlskog 2009). Dopaminergic dysfunction in the basal ganglia with parkinsonism has been suggested to be one rare explanation, while in many more patients, amiodarone-induced oscillatory movements as enhanced physiological tremor may be attributable to potentially reversible hyperthyroidism. (Ishida et al. 2010)

**Table 3:** Treatment options for common drug-induced tremor

Medication inducing tremor	Possible remedy
Beta-mimetics	Consider reducing frequency or discontinuing usage; alternatively, try longer-acting β-adrenergic agonists
Amiodarone	Evaluate for concurrent hyperthyroidism; if possible, decrease the dose to < 200 mg qd; consider adding a beta-blocker
Tricyclic antidepressants (e.g., amitryptiline)	Allow time to observe if tremors improve, or discontinue usage; consider using an SSRI. Add a beta-blocker if the tremor is disabling
Lithium	Check serum levels and lower the dose; switch to a different medication (such as valproic acid or lamotrigine); consider using a *β*-adrenergic antagonist
Metoclopramide	Discontinue usage and monitor the patient; consider alternative agents like erythromycin or domperidone for gastrointestinal motility disorders; monitor the patient for signs of parkinsonism and tardive movement disorders
Neuroleptics	Discontinue usage (if possible) or switch to an atypical neuroleptic with less antidopaminergic potency; add an anticholinergic or amantadine
Tacrolimus/Cyclosporine	Reduce the dose; switch to extended-release preparations; try another immunosuppressive drug
Valproate	Reduce the dose; switch to another antiepileptic; use β-adrenergic antagonists

Another significant cause of drug-induced tremor is the utilization of antidepressants. Research on selective serotonin reuptake inhibitors (SSRIs) and serotonin and norepinephrine reuptake inhibitors (SNRIs) indicates that approximately 20% of patients experience tremor (Diaz-Martinez et al. 1998). Clinical characteristics vary, but a frequency of 6–12 Hz has been observed in tremor associated with SSRI/SNRIs, due to elevated serotonin levels possibly impacting central dopamine transmission or stimulating the inferior olivary complex (Table 3) (Serrano-Duenas 2002). Tremor may also manifest during withdrawal, particularly with SNRIs (Baldwin et al. 2007). In certain instances, tremor may persist for weeks after discontinuation. Thus, it is advisable to reduce the dosage gradually. Tricyclic antidepressants such as amitriptyline can also induce tremor, possibly by increasing the central oscillator of physiological tremor (Raethjen et al. 2001). However, this tremor typically resolves over time with treatment. Lithium has the potential to cause substantial tremor, especially when the therapeutic dosage is exceeded. Although the precise prevalence remains unknown, it is widely believed that morbidity and non-compliance arising from the emergence of tremor present a significant side-effect (Baek et al. 2014; Burgess et al. 2001). One possible cause of low-amplitude 8–12 Hz tremor is increased activity of a central oscillator, and primidone or beta-blockers have demonstrated effectiveness in reducing tremor symptoms (Burgess et al. 2001). However, caution should be exercised due to the common occurrence of polypharmacy, which refers to the concurrent use of drugs that interact with lithium and increases the risk of lithium toxicity. (Dennison et al. 2011)

Anticonvulsants are known to cause tremors, with prevalence rates of up to 45% (Zadikoff et al. 2007). Valproate, in particular, has a prevalence rate of 14% based on a recent meta-analysis (Table 2) (Zhang et al. 2020). Tremor induced by anticonvulsants is characterized by low amplitude and simultaneous activity in agonist and antagonist muscles, occurring at a frequency of 10 Hz. Research suggests that women may experience a more severe tremor, and older individuals who have been on treatment for a longer duration may also be more susceptible (Alonso-Juarez et al. 2017). The severity of the tremor is positively correlated with higher doses and longer duration of treatment, although the association with total dose per kg or serum levels is still controversial (Alonso-Juarez et al. 2017; Hamed and Abdellah 2017). Beta-blockers are generally effective in treating tremor (Alonso-Juarez et al. 2017). Other anticonvulsants, like lamotrigine, have also been reported to cause tremor in a percentage of users, with rates as high as 10%. In fact, tremor has been reported in up to 25% of epilepsy patients treated with lamotrigine using accelerometry. Importantly, age and serum levels do not seem to correlate with lamotrigine-induced tremor. Here tremor is primarily voluntary, suggesting a possible involvement of cerebellar pathways and a potential association with cerebellar atrophy.

Antagonism at dopamine receptors is a mechanism common to several drugs used to treat psychosis and related disorders. However, it is important to note that these drugs can cause mostly asymmetric postural and rest tremor, known as drug-induced parkinsonism (DIP). The occurrence of DIP depends on factors such as the specific dopamine receptor antagonist, dosage, patient's age and gender, and family history (Mehta et al. 2015). Tremor is the initial symptom in around 35% of DIP cases, and patients treated with classical neuroleptics like haloperidol, thioridazine, and fluphenazine have a lifetime prevalence of up to 60% for developing tremor (Sethi and Zamrini 1990). The prevalence of DIP has decreased with the use of atypical neuroleptics like aripiprazole and olanzapine, and now it is observed only when drug use reveals the concurrent loss of dopaminergic neurons. However, since DIP can potentially be reversed over months to years, it is recommended to discontinue or switch to an atypical neuroleptic. In cases of DIP, tremor symptoms can be alleviated with anticholinergics or amantadine.

Metoclopramide is prescribed for gastro-esophageal reflux disease and gastroparesis, but it may lead to drug-induced movement disorders, such as parkinsonian resting tremor and essential-like tremor (Dennison et al. 2011). These disorders are believed to be caused by the cholinergic effects, possibly in combination with the dopamine receptor antagonism. Older patients and those with renal failure may be particularly at risk of developing metoclopramide-induced parkinsonism and tremor, so it is recommended to either avoid this medication or reduce the dosage (Sirota et al. 1986).

Higher doses of β-adrenoceptor agonists induce postural and kinetic tremor in 9% of patients treated with salbutamol and less with longer acting salmeterol (Ma et al. 2023). Prevalences may be higher when beta-mimetics are administered orally. The most plausible rationale is the involvement of peripheral mechanisms that activate β-adrenergic receptors in muscle spindles and fibres (Abila et al. 1985). This is also the underlying explanation for why nonselective beta-blockers effectively alleviate tremor.

Tremor induced by cyclosporine is generally of a mild to moderate nature and affects the entire body. While more noticeable when blood levels are elevated, some patients may experience tremor at lower doses so that no recommendations on dosage can be made (Gijtenbeek et al. 1999). Tacrolimus, which has been associated with tremor in reports of neurological toxicity following liver transplantations, particularly affects pediatric patients (Wijdicks et al. 1994). It specifically causes debilitating hand tremor that interfere with handwriting. In certain cases, reducing the dosage has proven to be beneficial in alleviating severe hand tremor. However, it is worth noting that some individuals may still experience mild and inconsequential tremor. (Wijdicks et al. 1994)

### Sensor based tremor detection

Tremor is now classified according to clinical characteristics (axis 1) and etiology (axis 2) (Bhatia et al. 2018). In the field of movement disorders, healthcare providers often face the challenge of objectively assessing the occurrence and severity of symptoms. Patients may not be able to provide reliable information about their symptoms, and external factors such as fatigue, stress, and medication can also influence the symptoms, as is the case with PD symptoms, dystonia, or tremor syndromes. Electrophysiology can serve as an extension of the clinical examination, particularly for physiologic tremor, primary orthostatic tremor, and functional tremor. However, it is also valuable in the clinical characterization of all types of. (Wardt et al. 2020)

In recent years, several devices, mostly including an accelerometer and gyroscope, have become widely available. Additionally, smartphones and watches allow for the measurement of motor skills, speech, and well-being through calls and questionnaires, regardless of time and location (Warmerdam et al. 2020). This monitoring can improve the quality of life for those affected, reduce the burden on healthcare systems, and track symptoms. Some even consider these measurements as digital biomarkers of diseases. (Tonges et al. 2022)

The use of unobtrusive recordings attached to the patients' bodies is particularly remarkable. Symptoms that are easy to track, such as tremor, can be easily measured, and valuable additional information can be collected. To assess tremor characteristics mobile phones, smart watches and other devices are used to assess tremor. (Fuchs et al. 2021; Vescio et al. 2021)

This can enable objective measurements leading to faster and more accurate diagnoses and aid in therapeutic decisions (Tonges et al. 2022; Lim et al. 2022; Deuschl et al. 2022). Quantitative analysis provided by sensors enables the measurement of different tremor parameters such as amplitude, frequency, and direction. This detailed analysis aids in understanding tremor patterns and can reveal subtle changes in disease progression over time.

In this regard, it has been shown that clinical severity can be correlated with inertial measurement units and electromyography recordings from affected individuals (Warmerdam et al. 2020). However, time series data not only allow for correlation with PD severity, but they can also be incorporated into clinical decisions and assist in, for example, facilitating adaptive DBS in patients with tremor. (Kleinholdermann et al. 2021, 2023; Biase et al. 2017; Cagnan et al. 2016) Integration of sensor-based measurements into telemedicine platforms allows for remote monitoring of tremor and facilitates teleconsultations with healthcare professionals. Moreover, these data help clinicians make more informed decisions regarding treatment strategies tailored to individual patient needs.

In the future, the application of machine learning algorithms may further improve the identification and treatment of movement disorders. (Kubota et al. 2016)

### Non-drug treatment for tremor using peripheral none-invasive medical devices

Non-drug therapy for tremor encompasses various approaches, including the use of aids like wristbands specially designed to reduce or control tremors. These therapy options offer an alternative or complementary approach to medication for tremor, especially for patients who do not benefit sufficiently from medications or experience side effects.

Specially designed wristbands with built-in sensors and stabilization technologies can help reduce tremors by applying targeted pressure or vibrations to affected areas (Dai et al. 2023). These wristbands can decrease shaking in hands or arms and improve daily activities such as eating, writing, or fine motor tasks (Dai et al. 2023). Apart from wristbands, other sensor-based technologies like gloves or stabilization systems can be used to control tremors (Mo and Priefer 2021). These systems detect patient movements and can counteract tremors effectively.

In addition to aids, physical therapy plays a role in non-drug treatment for tremor. Exercises aimed at improving muscle strength, coordination, and balance can help control tremors and enhance quality of life. (Shahien et al. 2023; Isaacson et al. 2020)

The psychological effects of tremor can significantly impact an individual's quality of life and emotional well-being. Tremors, whether caused by ET or PD, can lead to feelings of frustration, embarrassment, and social isolation. One of the primary psychological effects of tremor is the impact on self-esteem and confidence. Individuals with visible tremors may feel self-conscious about their condition, leading to decreased self-esteem and confidence in social interactions and daily activities. This can contribute to anxiety and depression, further affecting overall mental health.

Psychotherapeutic support can be beneficial for some patients in dealing with the emotional and social impacts of tremor (Lorenz et al. 2006). Techniques such as relaxation, stress management, and self-management strategies can improve quality of life. (Hopfner and Deuschl 2020; Schwingenschuh and Espay 2022; Lester-Smith et al. 2023)

Overall, non-drug therapy for tremor offers a range of approaches to alleviate symptoms and promote functional independence in patients. The choice of therapy depends on the type and severity of tremor, individual patient needs, and the availability of resources. A multidisciplinary approach involving neurologists, physiotherapists, occupational therapists, and psychologists can be highly beneficial in managing tremors effectively.

### Best practice recommendations and newest developments

- ET is distinguished from ET + . The latter is diagnosed when one of the following neurological symptoms ('soft signs') is present: slightly disturbed gait, questionable dystonic symptoms, discreet memory impairment, resting tremor, other discreet neurological abnormalities. The same therapy recommendations apply for ET and ET + .
- Besides the established therapies for ET (propranolol, primidone), topiramate (target dose ≥ 200 mg daily) is classified among the recommended medications. All three medications have different side effect profiles, which are crucial for the indication.
- The effectiveness of electromyographically guided botulinum toxin has been demonstrated with a favorable side effect profile for dystonic arm tremor.
- Magnetic resonance imaging-guided focused ultrasound (MRgFUS) provides a new, non-invasive, lesioning therapy for unilateral treatment of medication-resistant ET and, with insufficient evidence to date, also for Parkinsonian tremor.
- Specialized physiotherapy with education, movement retraining, and long-term focus on self-management has been shown to be effective in the treatment of functional tremor.
